# Defining Optimal Post-prison Care for Those With Psychosis: A Delphi Study

**DOI:** 10.3389/fpsyt.2021.760904

**Published:** 2021-10-22

**Authors:** Paul L. Simpson, Stella Settumba, Armita Adily, Bianca Ton, Tony Butler

**Affiliations:** ^1^School of Population Health, University of New South Wales, Sydney, NSW, Australia; ^2^National Drug and Alcohol Research Centre, University of New South Wales, Sydney, NSW, Australia

**Keywords:** prison, care, psychosis, offending, treatment, Delphi

## Abstract

**Background:** Early treatment (considered as early contact with community mental health services) and treatment retention are associated with reduced reoffending among those with a previous diagnosis of psychosis, yet the attributes of care required to best achieve this is largely unexplored for people with psychosis leaving prison. This study sought consensus from a sample of experts and consumers regarding the attributes of an “optimal model of care” for those with a prior episode of psychosis leaving prison in New South Wales, Australia.

**Methods:** A Delphi method was used, which involved establishing a consensus from a panel of 25 experts and consumers. Following three meetings, 34 model of care attributes and 168 attribute levels were generated for two rounds of online scoring. All attributes and levels were included in the final model if they scored “very important” or “extremely important;” or if the attribute was agreed on by 70% or more of participants. The participant retention rate across scoring rounds was 96% for Round 1 and 84% for Round 2, where consensus was reached. Two “member checking” procedures were undertaken to enhance the integrity of findings: a model “stress test” and an online consumer poll.

**Results:** Thirty-two attributes and 72 attribute levels were included in the final model across four components: pre-release care planning and coordination; treatments in community; diversion from prison; and evaluation. Member checking endorsed a person-centered approach with carers and peer-support central to care.

**Conclusions:** Participants agreed that an optimal model of care should involve a specialized team who works independent of community health service teams to directly deliver certain treatments and services while helping consumers to access external social an economic supports and services.

## Introduction

Incarcerated populations are consistently recognized as having some of the highest rates of mental illness of any population group (1–4). Studies consistently illustrate that psychosis is associated with criminal convictions, particularly for violence (5–9), however, reported associations depend on study design, sample size, different treatment and legal practices in a jurisdiction, and resources for treatment and support.

In 2016, we undertook a population-based data-linkage study to examine the association between psychosis and offending in the Australian state of New South Wales (NSW) which houses 33% of the nation's incarcerated population (10). Cases were all individuals diagnosed with psychosis between 2001 and 2012 (*n* = 86,461). For each case, two controls were matched by age and sex (11). In addition to endorsing the association between a diagnosis of psychosis and criminal convictions, including for violence, we found that increased treatment (defined as contact with community mental health services) (12), retention in treatment (13), early commencement of treatment following an offense (11), and diversion by the courts into treatment were associated with reduced reoffending (14). These findings indicate that treatment in the community rather than punitive sanctions is important, yet the attributes of care required to best achieve reduced reoffending is largely unexplored.

Community-based models of care for people with psychosis have tended to reflect either a “merged model” where forensic specialists work within community mental health service teams, or an “independent model” where forensic specialists work as a team independent of community health service teams (15). Regarding the later, the “Assertive Community Treatment” (ACT) model has become one of the prominent independent models (16). The key principles of ACT include outreach, direct delivery of treatments and services in the community including the patient's home by a specialized team, holistic and integrated services, and continuity of care (16–18). Although model fidelity concerns exist among some, there have been numerous adaptations of ACT including for forensic populations (17).

Complex inquiries regarding an optimal model of care for a specific population can perhaps be best answered by a panel of “experts,” including service providers and planners. In recent years, a focus on equity-orientated priority setting has emerged whereas input into decision-making have centered on the inclusion of diverse stakeholders rather than single-specialty experts (19, 20). Most studies have used the Delphi method to determine expert (21–23) or consumer (24, 25) consensus on psychosis care. A number of Delphi studies have included both consumer (and carer) and experts, with several using separate panels (26, 27). Very little Delphi studies include single panels made up of consumers and other experts such as researchers and clinicians who deliberate together to provide input into the development of the Delphi scoring survey (28). Additionally, a paucity of studies have sought consensus for models of care for forensic populations (15, 23).

The objective of this study was to determine the key attributes of an “optimal post-prison model of care” for those with a prior episode of psychosis, using the Delphi method to establish a consensus among experts and consumers (hereafter includes consumers as experts). The study was requested and funded by the NSW Department of Communities and Justice (DCJ) at a time when both the DCJ and NSW Health Ministry were implementing two independent pilot programs for people with serious mental illness leaving prison.

## Methods

**Figure 1:**
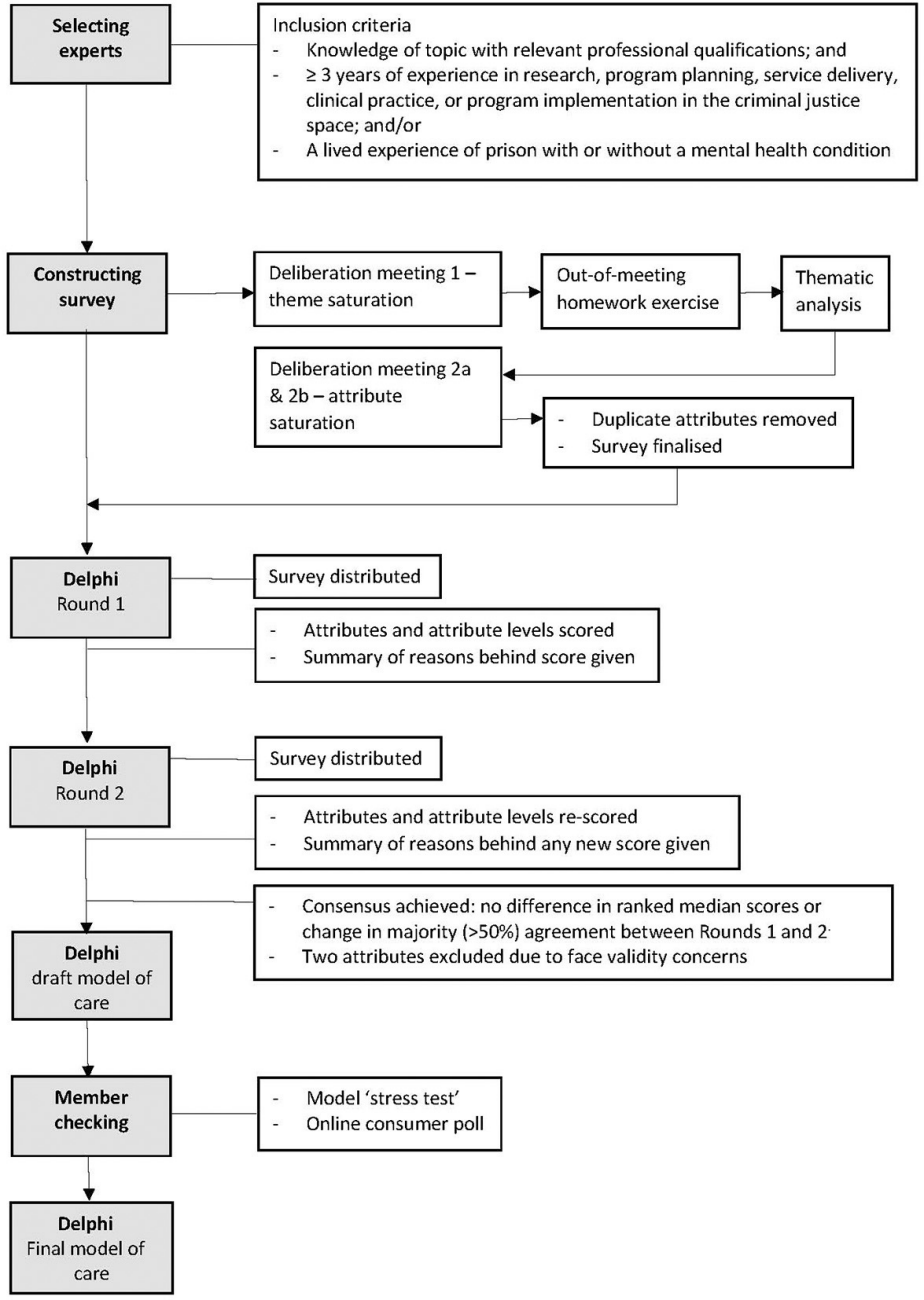
Stages of the Delphi study.

The Delphi method involved three rounds of deliberation by experts designed to inform survey construction, followed by two rounds of survey distribution to experts where model of care (hereafter “the model”) attributes and levels (i.e., attribute dimensions) were scored and commented on, to establish a convergence of opinion (Figure 1). Convergence was assessed by examining the similarity or central tendency of participants' responses to each question across rounds. Convergence was used as a measure of agreement or consensus (29).

### Selection of Experts

There are no universally agreed criteria for the selection of experts for Delphi studies. Recent literature has highlighted the need to examine the assumptions and values that may influence expert input on contested topics, as “determining who should have a voice in building future scenarios can be marred with politics, conflicts, and competing interests” (30). Such disagreements among experts can emerge regarding structural uncertainties (related to system boundaries) and epistemic and normative differences amongst participants. Therefore, it may be necessary to ensure an even and diverse spread of experts across disciplines and policy/portfolio priorities attached to agencies/organizations in which experts are affiliated with to minimize bias. To further offset any accusations of favoritism, bias, and subjectivity, a clear and transparent participant selection criterion is essential (30).

For the present Delphi study, the inclusion criteria was persons with knowledge and insight into the topic with relevant professional qualifications, and at least 3 years' experience in research, program planning, service delivery, clinical practice, or program implementation in the criminal justice space, and/or with a lived experience of prison with or without a mental health condition. Using these criteria, a preliminary list of prospective experts was compiled in consultation with Corrective Services NSW (CSNSW), Justice Health and Forensic Mental Health Network (JH&FMHN) staff, and other key experts. JH&FMHN is part of the NSW government's Ministry of Health who are responsible for providing health services to those in contact with the NSW criminal justice and forensic mental health systems, and CSNSW is responsible for NSW prisons and the supervision of people on parole and community-based orders. This list consisted of 13 experts from CSNSW (*n* = 5), JH&FMHN (*n* = 4), non-government experts (*n* = 2), Mental Health Branch of the NSW Ministry of Health (*n* = 1), and the NSW Mental Health Review Tribunal which makes decisions on the management and treatment of forensic patients (*n* = 1). To achieve a sample size of at least 20 experts, and assuming a response rate of 50%, the research team expanded this list to 40 prospective experts. The selection of the additional 27 experts was informed by diversity considerations and to ensure consumer/consumer advocate voices were represented. The final invitation list consisted of experts from CSNSW (*n* = 9), JH&FMHN (*n* = 9), Consumer/Consumer support NGO (*n* = 5), NSW Mental Health Review Tribunal/Mental Health Commission (*n* = 4), non-government expert/scholar (*n* = 4), NSW Ministry of Health (*n* = 3), consultant forensic psychiatrists (*n* = 4), NSW Police (*n* = 1), and a Chief Magistrate (*n* = 1). “The Consumer/Consumer support NGO group consisted of three participants with lived experience of prison (one of whom had a lived experience of psychosis, and another who was affiliated with a NGO that advocates for improvements in the criminal justice and mental health systems), one participant with lived family experience of long-term mental illness, and one participant affiliated with a NGO that provides support and advocacy for people in and leaving prison.”

### Survey Construction

Two deliberation meetings informed survey construction, the outcomes of Meeting 1 were used to identify thematic components of a model of care, and the outcomes of Meeting 2 were used to identify attributes and levels associated with each thematic component. Due to Covid-19 restrictions, meetings were convened online using a video conferencing platform. The facilitators of the meetings were independent (to the research team and stakeholder agencies) to prevent agenda bias.

#### Meeting 1—Theme Saturation

The objective of the first meeting was to reach saturation of thematic components of the model. Following introductions and a project background presentation, a “speed geeking” exercise was conducted. This involved four pre-selected experts presenting for 10 min each to participants in four small virtual breakout rooms. Presentations included models of care used in other jurisdictions (1), challenges and considerations to successful models of care in NSW (2), and a consumer's perspective of care received for serious mental illness inside and upon leaving prison (1). After their presentation and Q&A, presenters moved to a new virtual room to present. This was repeated until all groups had heard from all the expert presenters. A research team member was present at each breakout room and took notes. Using an online platform (Stormz), participants were asked to post what they thought to be the key elements to consider in a model of care, following which a list of themes relating to the model of care were identified. These were subsequently refined and scored during an online out-of-meeting homework exercise for participants. Meeting 1 input in Stormz included 84 ideas. A number of these ideas were placed in what was identified by the group as draft themes. This input together with the breakout room notes were thematically analyzed by one member of the research team (PS), and cross checked two other research team members (SS, TB). Four broad principles of the model and five specific themes were identified. A potential theme was allocated as a principle if it represented a foundational idea or approach which most participants saw as being important at every aspect of the model or consumer's journey.

#### Meeting 2—Attribute Saturation

At the second meeting, attributes and levels were generated for each theme. An attribute was defined as a characteristic of the theme which can take on various dimensions or levels. For example, “assessment” can be a characteristic of the theme “care planning” which may have various levels such as “clinical assessment,” “reoffending risk assessment,” and “socio-economic needs assessment.” Meeting 2 was convened in two separate sessions due to participant availability (Meeting 2a and 2b). At Meeting 2a, theme attributes were listed by participants (*via* Stormz). Participants then delved deeper into specific themes to exhaust divergent ideas on these attributes *via* small breakout groups. As in Meeting 1, a research team member was present at each breakout room and took notes on attributes and levels put forward by the group. These notes together with attributes listed in Stormz saw participants contribute a further 190 attribute and level ideas. With no new attributes generated at the end of meeting 2b, attribute saturation was assumed for most themes.

Following the removal of duplicates, a total of 34 attributes and 168 attribute levels were generated from Meetings 2a and 2b. These were then grouped according to the model's thematic components derived from Meeting 1 and used to construct an online survey using Qualtrics software. Three draft versions of the survey were reviewed by the research team before a final survey was distributed.

### Delphi Scoring Rounds

In Round 1 scoring, participants were asked to score each attribute and/or level using a Likert scale response format ranging from 0 (“not important”) to 5 (“extremely important”). Importance is described as the consideration given to an attribute to be included in the model. Some questions were in the form of agreement statements where for example participants were asked “Do you agree/disagree with the following statements? [‘Yes' or ‘No'].” For these questions, an overall percentage agreement by the group was determined. Participants were also requested to provide a short explanation for each score against each attribute. Once Round 1 surveys were completed, a median score for the group was calculated for each attribute and level that was scored using a Likert scale response format. For attributes that were scored using an agreement statement format, the percentage of participants who indicated “Yes” and “No” was calculated. Following this, all attributes within each theme were listed from highest to lowest median score or agreement percentage before being sent back to participants for Round 2 scoring. To ensure that all participants' views were considered by the other experts when scoring in Round 2, a summary of the reasons given by participants when assigning scores for Round 1 was provided against each attribute.

In Round 2, the list of attributes and summary comments per attribute were returned to each participant, indicating their individual response from Round 1 and how this compared with the overall median and percentage scores of the group. Each participant was then given an opportunity to reconsider the importance of each attribute and re-score each attribute. Participants were asked to provide a comment against any new score that differed by two points from the group median or if they changed an agreement statement response from “Yes” to “No” or from “No” to “Yes.”

This Delphi process is typically repeated in subsequent voting rounds until convergence of group opinion or “consensus” is achieved. Consensus is defined as there being no difference in ranked median scores or change in majority (>50%) agreement in the last two rounds of scoring. Consensus is typically reached by Round 3. All attributes and levels included in the model scored “very important” or “extremely important”; or if the attribute was agreed on by 70% or more of participants.

### Member Checking

Member checking, also known as participant validation, is a technique for exploring the integrity of findings and involves returning the data to participants to check for accuracy or identifying substantial omissions. Member checking can also occur by assessing the extent to which the data can be corroborated by other available data or information from stakeholders on the topic at hand (31). Two “member checking” procedures were undertaken to assess the integrity of findings: a model of care “stress test” with experts and an online consumer poll. These activities aimed to identify model attributes or attribute levels that were overlooked in the final model arrived at or attributes that were seen as questionable in terms of preventing model effectiveness.

Delphi experts were invited to attend a final online stress test meeting which involved generating reasons for the model's failure and allows for all participant voices to be heard and their input reported. Fourteen participants attended the “stress testing” meeting and created 47 idea cards and 17 comments entered again in the online platform Stormz.

An online consumer poll was also undertaken with people in the wider community with lived experienced of prison and their family members to hear their views on ways to support people leaving prison with a past episode of psychosis. This was achieved using a survey link posted on the Facebook group “Australian Advocate for Prisoners and their Families” which has 14,000 members. The survey asked participants to rate the importance of 24 model of care attributes. Two open-ended questions were also posed to capture participant thoughts on attributes not listed and ways to help people stay engaged with support and treatment. Two Delphi experts with the lived experience of prison, who are administrators of the Facebook group, facilitated data collection. Thirty-six people contributed to the poll: 27 completed most or all items, and 25 left responses to the open-ended questions.

### Ethics Approval

Ethics approval was obtained from the University of New South Wales Sydney Human Research Ethics Committee (HC200339).

## Results

A total of 25 experts participated in Meeting 1 (theme saturation), with 22 (88%) attending either or both Meetings 2a and 2b (attribute saturation). The participant retention rate for scoring was 96% for Round 1 and 84% for Round 2, where convergence of opinion was reached. Participants were from JH&FMHN (28%), CSNSW (16%), consumer/consumer support NGO (16%), consultant forensic psychiatrist (12%), NSW Mental Health Review Tribunal and former Mental Health Commissioner (8%), a non-government expert/academic (8%), NSW Ministry of Health (8%), and the NSW Police (4%).

Four model of care principles were identified: care integration and coordination; person-centered approach; evidence-based approach; and commitment, understanding and resourcing by government (Table 1). Four model themes where identified: pre-release care planning and coordination; treatments in community; diversion from prison; and evaluation (Table 1). A fifth theme that was originally identified—social and economic supports—was reconfigured as attributes under the pre-release care planning and treatment themes.

**Table 1:** Principles to underpin, and thematic components of, an optimal model of care for people with psychosis leaving prison, as identified by participants.

**Model of care principles and thematic components**	**Definition**
**Model of care principles**
1. Integrated services and coordination^a^	The organization and management of services so that people get the care they need, when they need it, in ways that are user friendly, achieve the desired results and provide value for money.
2. Person-centered approach^b^	The individual is placed at the center of the service and treated as a person first. Focus is on the person and what they can do, not their condition or disability. Considers the individual's life experience, age, gender, culture, heritage, language, beliefs, and identity.
3. Evidence-based approach	The integration of research evidence with clinical and stakeholder expertise and patient values to achieve optimal outcomes at each stage of the model of care pathway.
4. Commitment, understanding, and resourcing by government^b^	Ongoing government commitment and resources should be directed at understanding and addressing the gaps in care and supports in the community to ensure systems are well equipped to assist people with psychosis who leave prison achieve optimal outcomes.
**Model of care thematic components**
1. Pre-release planning and coordination^a,b^	Assessment, integrated care planning development, and coordination before release from prison.
2. Treatments in community^a^	Integrated, comprehensive, and specialized treatments/services that address the psychological, medical, and social needs of the person.
3. Diversion away from prison^a^	Diverting people away from the criminal justice system following relapse/decompensation/system failure.
4. Evaluation	Evaluation of the design, implementation, and outcomes of an ongoing model of care to determine the relevance and achievement of objectives and the efficiency, effectiveness, impact, and sustainability of the model.

a *Identified as important in consumer online poll*.

b *Reinforced as important in model stress test to prevent model failure*.

Of the 34 attributes and 168 attribute levels, 32 attributes and 72 attribute levels were included in the model (Table 2). Two rounds of scoring were conducted before consensus was reached for all attributes and levels except for two “diversion from prison” attributes. These related to the type of “offense committed” or “parole condition breached” that would prevent someone from automatically be considered for diversion from prison by the courts. Seventeen participants provided comments for these two attributes suggesting that most participants were reluctant to commit to a particular offense type or parole condition breach as criteria for diversion. Following discussion by the research team, it was decided not to ask participants to re-score these two attributes in a Round 3 survey due to face validity concerns and the unlikely prospect of reaching a consensus for these attributes. Consequently, these two attributes were excluded from the final model (see Supplementary Material A).

**Table 2:** Optimal model of care attributes and levels scored by participants as important.

**Thematic component**	**Attribute**	**Level**	**Rating of importance (from 1 to 5; 5 being extremely important) or percentage of participants who agreed with attribute statement**
**Pre-release planning and coordination**	Assessment	Clinical assessment	5
		Social and economic assessment	4
		Reoffending risk assessment	4
		Demographic (e.g., gender, culture) assessment	4
	Assessment timing	Upon prison entry, repeated at regular intervals	5
	Exclusion criteria	Nil, case-by-case basis	100%
	Stakeholder involvement at planning stage	NGO/Aboriginal Community Controlled Health Organization	5
		Government housing provider	5
		Mental Health Review Tribunal	5
		Justice Health and Forensic Mental Health Network	4
		Community Corrections	4
		Consumer/carer/family member/guardian/peer^a,b^	4
		Correctional center personnel	4
		Police	4
	Stakeholder lead at planning stage	Justice Health and Forensic Mental Health Network	4
		Consumer/carer/family member/guardian/peer^a,b^	4
		Local Health District (NSW Ministry of Health)	4
		Establish a new independent specialized service/team for this model of care	4
	Case manager to operationalize plan	Case manager affiliated with a new independent specialized service/team created especially for this model of care	71.4%
	Consumer engagement	Undertake motivational work in prison to foster consumer engagement in care planning before release	4
		Address engagement barriers that may occur in prison (e.g., mental health related stigma, consumer confidentiality issues, timely access to mental health services, fear of “Risk Intervention Team” practices (e.g., safe cell/isolation)	4
		Mandate care plan if necessary (e.g., treatment order)	4
	Information sharing between agencies/organizations	Information sharing agreement with confidentiality provisions^b^	95.2%
**Treatments in community**	Case management approach	Cases managed by a specialized team delivering treatments with brokered service options	85.7%
	Team composition	Psychiatrist	5
		Nurse	4
		Psychologist	4
		Social worker	4
	Treatment access	Tele-health service using audio-visual link on computer	4
		Transport support to attend treatment/service^b^	4
	Teams works with consumers' informal support network		4
	Team leader	Psychiatrist	5
	Contact frequency with consumer	1–2 times per week	90.5%
	24-h crises response^b^	*Via* general crises line	4
	Team closely involved in hospital admission and discharge decisions		4
	Case load	10 or less cases per case manager	71.4%
	Treatment type	Psychological support/counseling	5
		Drug and alcohol support	5
		Independent living skills support	5
		Pharmacotherapy	5
		Social and economic supports^b^	5
		Residential rehabilitation	4
	Treatment delivery	Psychological support/counseling by new independent specialized service/team	76.2%
		Drug and alcohol support by new independent specialized service/team	71.4%
		Independent living skills support by NGO/Aboriginal community-controlled health organization^a^	100%
		Pharmacotherapy by Local Health District (NSW Ministry of Health)	81.0%
		Social and economic supports by NGO/Aboriginal community-controlled health organization	95.2%
		Residential rehabilitation by NGO/Aboriginal community-controlled health organization	100%
	Treatment location	Treatments/services should be split 50/50 between outreach in community and based at the office location of the treatments team	71.4%
	Supervision of medium to high reoffending risk	Case manager who implements the consumer's care plan (if case manager is not Community Corrections Officer)	81.0%
	Forensic mental health expertise/training	Psychiatrist	5
		Nurse	5
		Psychologist	5
		Substance abuse specialist	4
		Probation and parole/CCO	4
		Aboriginal health practitioner	4
		Social worker	4
		Social support service provider (NGO)	4
		Peer support worker	4
		Vocational/training specialist	4
		Housing provider	4
**Diversion from prison**	Diversion from prison is the default option for all consumers until the prosecution convinces the court otherwise		85.7%
	A treatment and care plan should be made available for those not diverted and return to prison		100%
	Consumers diverted from prison should have their care plan reviewed		100%
	Court diversion should be available to all consumers (regardless of residential address)		100%
**Evaluation**	Effectiveness in managing psychosis and comorbidity		5
	Effectiveness in reducing reoffending		4
	Consumer satisfaction		4
	Social and economic outcomes		4
	Health economics evaluation		4
	Successful integration of services		4

a *Reinforced as important in model stress test to prevent model failure*.

b *Identified as important in consumer online poll*.

For the model stress test activity, participants did not identify any attribute of attribute level that could explain the model's failure. Instead, participants reiterated the importance of specific model principals or attributes, such as the model must be person-centered, encompass a trauma informed care and recovery approach, and that carers and peer support workers be central to care plan development and treatment decisions (see Table 1 footnotes). Findings from the consumer poll reinforced model thematic components and specific model attributes. However, poll participants did not comment on evaluation and evidence-based model themes as they were asked about the care components of the model and not on data aspects of a model (see Supplementary Material B for results).

## Discussion

In this Delphi study, a total of 32 attributes and 72 attribute levels across four themes were identified by experts to achieve an optimal model of care for people leaving prison who have experienced psychosis. Participant retention rates and level of input indicated a high level of their engagement in the process. Typically, consensus is reached in Delphi studies after three or more scoring rounds. For this study, consensus was reached after two scoring rounds. Although this may be interpreted as having a dominant scoring group or there being no contentious issues in this Delphi process, a probable explanation may lie in the scoring and attribute refinement activities participants engaged in as part of the online meetings. Thus, less relevant, or contentious model attributes were excluded from the scoring survey. Member checking processes overall reinforced the identified model of care principles and attributes, particularly underscoring the principal of a person-centered approach and involvement of peers.

A study limitation is potential affiliation and epistemic bias among experts. Although the participants' characteristics for the present study showed that a fairly even spread of affiliations and expertise was achieved, a higher proportion of participants affiliated with JH&FMHN (28%) took part compared to participants affiliated to CSNSW (16%) and consumer representatives (16%). To explore this potential bias, we examined the responses to specific questions which allowed respondents to select a specific agency to lead or undertake a responsibility attached to a model of care attribute. For example, responses to the attribute “consumer care planning lead” showed that 50% of CSNSW participants and 43% JH&FMHN participants gave the highest scores to agencies that they were not affiliated to theirs, and 50% of CSNSW participants and 57% JH&FMHN participants gave highest scores to the agency to which they were affiliated with (with some also scoring other agencies with the same highest score). These responses suggest participants' decisions can be informed by considerations beyond organizational allegiances. For the question: “who should supervise individuals assessed as medium to high reoffending risk,” 100% of CSNSW participants selected Community Corrections Officers (CCOs), and 100% of JH&FMHN participants selected the “case manager who implements the care plan” of which 57% of JH&FMHN participants selected that this case manager should not be JH&FMHN affiliated. Additionally, three participants not affiliated to either a DCJ agency or JH&FMHN selected that supervision should be undertaken by a CCO. It is difficult to determine whether these responses indicate (i) affiliation bias (for or against CSNSW), (ii) an objective and critical assessment of the attribute level, and/or—for participants preferring CCO supervision—(iii) current legislation requiring CSNSW to supervise this group and the substantial legislative reform required if this was altered to a non-CSNSW case manager. In reviewing, the comments provided by participants for this model attribute, factors (ii) and (iii), rather than affiliation bias, seem more likely to inform participant considerations. Although it is difficult to rule out potential affiliation and epistemic bias among experts as a study limitation, participants responses suggest that their decisions were informed by considerations beyond organizational allegiances.

Another limitation is that although experts included people with a lived experience of prison with or without a mental health condition and lived family experience of long-term mental illness, only one participant had both a lived experience of prison and psychosis. After consulting with our networks, it was assessed that it would likely be difficult recruiting people with both a lived experience of prison and psychosis. As reported in a review of Delphi studies (27), recruiting some minoritised consumer groups (e.g., specific refugee groups) can be difficult as relevant mental health advocacy and support NGOs for these groups are rare or do not exist. In response, we ensured that people affiliated with NGOs that provide support and advocacy for people in and leaving prison or for people with a lived experience of mental health systems were recruited. As people with serious mental health problems are overrepresented among incarcerated populations (2), mental health care of this population is typically a key advocacy area for the former NGOs.

A paucity of previous studies exists on stakeholder priorities of model of care attributes for forensic populations with psychosis with which to directly compare our findings. Nonetheless, many of the model attributes identified by participants in the present study are consistent with independent model attributes (15) such as the ACT (17), albeit with one notable departure. Although a high-fidelity ACT model instructs that an independent specialized team has full responsibility for, and directly provides, all treatments/services including psychiatry services, counseling, housing support, substance abuse treatment, and employment and rehabilitative services (16, 17), a hybrid case management approach was preferred by participants. This hybrid approach includes a specialized team directly delivering certain treatments/services while helping consumers to access and navigate external services including general medical practitioners, allied health professionals, pharmacists, Aboriginal health practitioners, and housing and income support providers (i.e., traditional brokerage model of case management). While some participants preferred the ACT model in terms of ensuring treatments and services align to the model's principle of care integration and coordination to prevent consumers “falling through the cracks,” most saw the hybrid model as a more appropriate balance given that individual consumer's level of need can vary over time and does not preclude an individual developing some level of independence. In this sense, the hybrid model was seen as better respecting and fostering consumer autonomy and independence and aligning to the model of care principle of being person-centered.

One issue often raised concerning the Delphi method is the generalizability of resulting outcomes. Sample size and sampling method are cited as two design features that can impact on the generalizability of Delphi findings. Given sample sizes of Delphi studies have ranged from 10 to 1,000, the sample size of the present study may be assessed as small or moderately sized. Additionally, we did not employ a random sampling method as this was not feasible to capture the diverse characteristics of our experts. As such, this likely reduces the generalizability of the results beyond NSW or Australia. However, we contend that the thematic components of the model and specific model attributes are likely to be generalizable to other jurisdictions, albeit with some modification based on the local resource, legal, and demographic realities. Modification to models of care for forensic populations. Despite fidelity concerns in modifying a model, there have been numerous adaptations of the ACT for example, including for forensic patients, to address the concerns with applying an “one-size-fits-all” model to different populations (17). The present Delphi study was commissioned by the DCJ to address the local NSW context. We noted previously that two separate pilot models of care were being implemented by DCJ and NSW Health Ministry before the results of the current study were released, and propose that future research examine the extent to which pilot model attributes align with the findings presented here.

## Conclusions

Participants were asked to envisage an optimal model of care without having their thinking impeded by existing social or structural challenges. Participants agreed that an optimal model of care should include an independent specialized team directly delivering some treatments/services while helping consumers to access external services. Such a model may need to traverse traditional systems boundaries, require policy and legislative reform, as well as undergo rigorous evaluation to ensure iterative model of care enhancements as required. This may entail resource and budgetary implications for government. Studies assessing the ACT model for forensic populations indicate that sustainability can be a significant challenge with teams funded with time-limited seed money, and that once these funds are exhausted, teams were disestablished, or services diluted (17). These issues were acknowledged by participants and underscore the importance of the model's principle of commitment, understanding, and resourcing by government.

## Data Availability Statement

The original contributions presented in the study are included in the article/Supplementary Material, further inquiries can be directed to the corresponding author/s.

## Ethics Statement

The studies involving human participants were reviewed and approved by University of New South Wales Sydney Human Research Ethics Committee (HC200339). Written informed consent for participation was not required for this study in accordance with the national legislation and the institutional requirements.

## Author Contributions

PS, SS, AA, and TB contributed to the conception and design of the study. PS and SS performed the analysis. PS wrote the first draft of the manuscript. SS, AA, BT, and TB made revisions to the draft manuscript. All authors read and approved the submitted version.

## Funding

This work was supported by a grant from the New South Wales Department of Communities and Justice. The authors declare that the research was designed, conducted, and findings analyzed and interpreted without input or influence by the funder. PS reports receiving support from a National Health and Medical Research Council grant APP1129816. TB reports receiving support from a National Health and Medical Research Council grant APP1124299. SS reports reviving support from the University of New South Wales. AA reports receiving support from a National Health and Medical Research Council grant APP1057492. BT reports receiving support from a grant from the New South Wales Department of Communities and Justice.

## Conflict of Interest

The authors declare that the research was conducted in the absence of any commercial or financial relationships that could be construed as a potential conflict of interest.

## Publisher's Note

All claims expressed in this article are solely those of the authors and do not necessarily represent those of their affiliated organizations, or those of the publisher, the editors and the reviewers. Any product that may be evaluated in this article, or claim that may be made by its manufacturer, is not guaranteed or endorsed by the publisher.
