# Supplementary material for: Defining Optimal Post-prison Care for Those With Psychosis: A Delphi Study
Source: Front Psychiatry. 2021 Oct 22;12:760904. doi: 10.3389/fpsyt.2021.760904 (PMC8569300; doi:10.3389/fpsyt.2021.760904)
Supplement: Supplementary file 1 [file Data_Sheet_1.docx]

**Supplementary Material B – Member checking activity results**

Results of the two member checking activities: a model of care stress test and an online consumer poll.

**1. Model of care stress test**

Fourteen experts and consumers participated in the ‘stress testing’ meeting and created 47 idea cards and 17 comments. Four main model of care ‘failure themes’ were identified.

1. Lack of resources and funding
2. Lack of governance and interagency cooperation
3. High reoffending rates due to being a high-risk population, and ineligible persons being referred to model of care
4. Inadequate consultation and involvement of consumers and carers

Other issues raised included that it is important that the model be person-centred, encompass a trauma informed care and recovery approach, and that carers and peer support workers be central to care plan development and treatment decisions.

**2. Online Consumer Poll**

Thirty-six people participated in the online poll from a Facebook group of over 14,000 members representing those with the lived experience of prisons in Australia: 27 completed most or all items, and 25 left responses to the open-ended questions. Responses to the 24 items were grouped into four model of care themes: (i) pre-release planning and coordination; (ii) treatment; (iii) social and economic supports; and (iv) other important model attributes.

**2.1 Pre-release planning and coordination**

The top three most important attributes for the theme ‘pre-release planning and coordination’ were ensuring that pre-release planning occurred, improved communication between different support and treatment services, and ensuring that the person has access to their own prison health records when returning to the community.


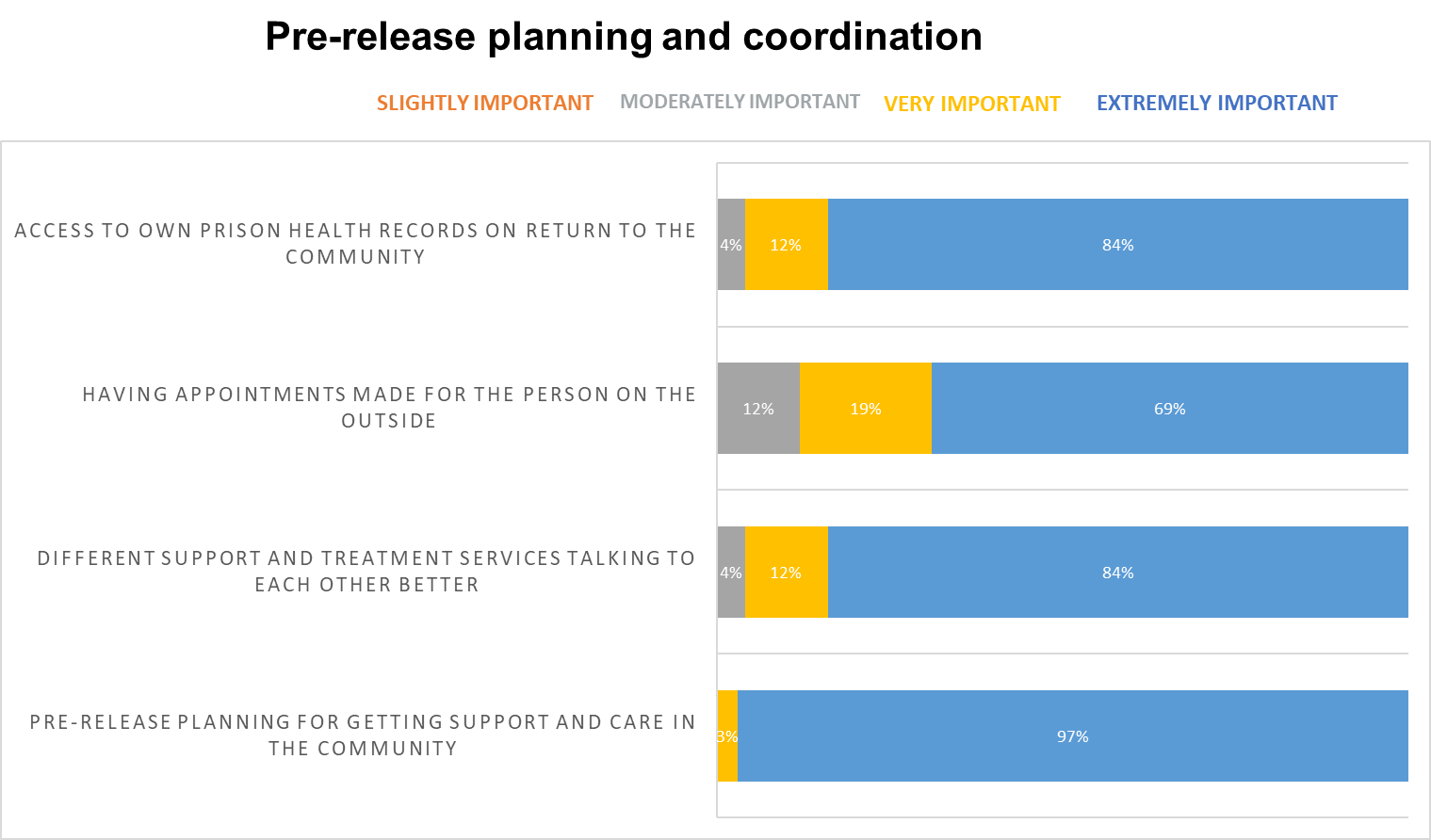


**2.2 Treatment**

The top three most important attributes for the ‘treatment’ theme were 24-hour access to mental health care, having not just medications but also other kinds of help, and access to culturally safe mental health care such as an Aboriginal Health Service.

***
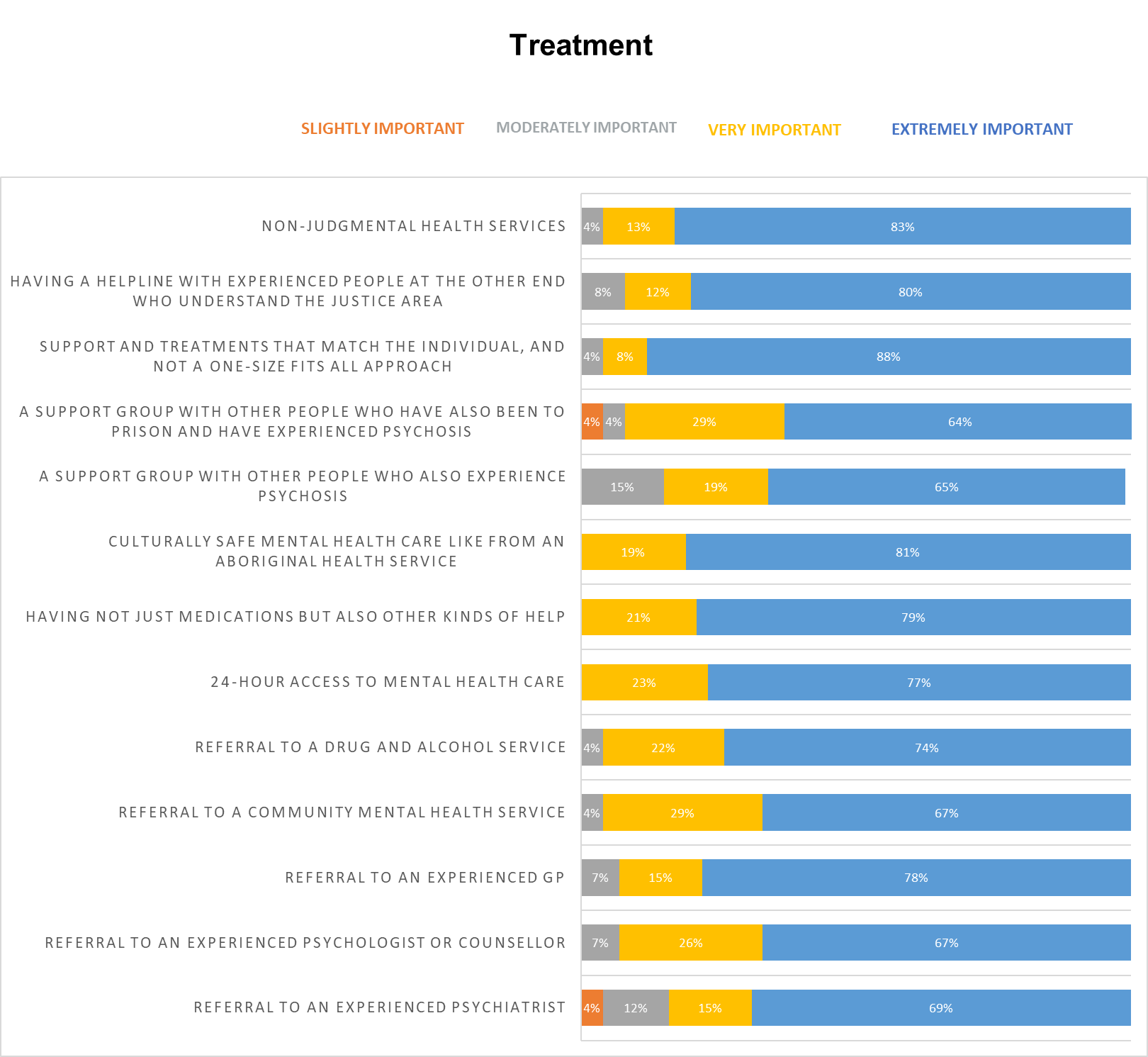
***

**2.3 Social and economic supports**

The top three most important attributes for the ‘social and economic supports’ theme were having somewhere to live on release that is stable and affordable, unemployment support when needed, and contact information for all the services that can help (e.g., mental health, lifeline, drug and alcohol, GP).


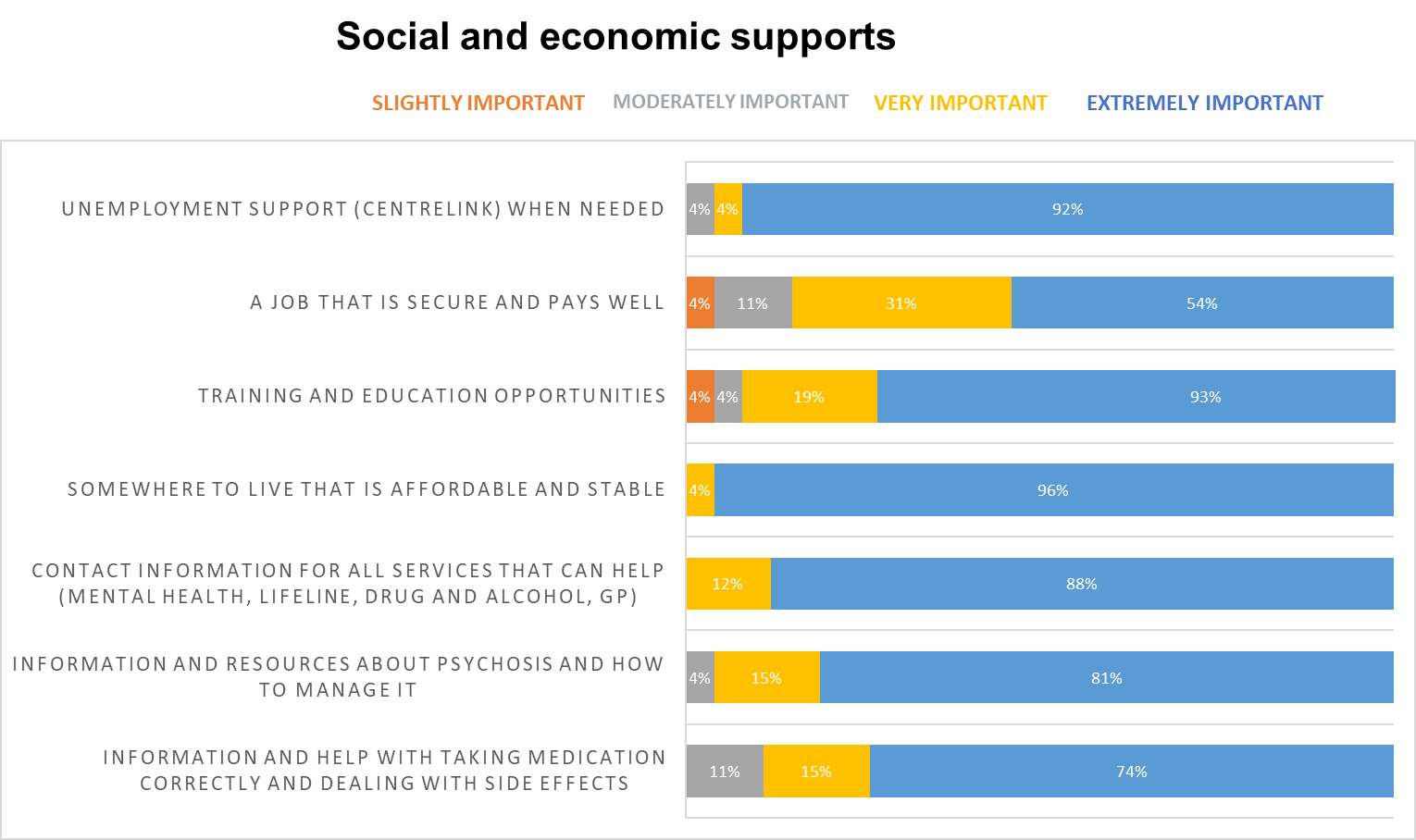


**2.4 Other important model attributes**

When asked to list any other care attributes that were important and not covered by the previous 24 items, as well as what might help people stay engaged to support and treatment, various responses were provided, with three themes identified: peer support; treatment/service accessibility; and “what will not work”. Qualitative data is presented below for each theme.

*Peer support*

“I think a buddy system could help within this group. They can be paired up with a suitable friend from the group & give 24-hour support. Such as a call if feeling stress, anxiety, scared of relapses, help with filling in forms, confidence building together, work on skills for empowerment, etc.”

“Recovery from any form of trauma requires behaviour change and support. Like any change it needs understanding and sometimes that is best from others who are in recovery and have lived through and survived the experience.”

*Treatment/service accessibility*

“For services to make the contact, not leaving it up to the client. Services to do outreach and follow up.”

“Coming out of gaol with no one to come out to is daunting so transportation other than public transport (tend to think people are staring); expensive taxis to get from gaol to wherever we need to go and help getting ourselves back together.”

*“What will not work”*

“Don’t chase them away with routines they resent already. Fit an individual programme to the individual.”

“I think there ought to be incentives to continue rather than punishments for not doing so. We tend to be a bunch that dislikes authority and be instructed on what to do and when to do whatever it may be. However, if these choices are made by us and again given there may be some kind of incentive in place, I think continued participation projections can be far greater.”
