# Supplementary material for: Defining Optimal Post-prison Care for Those With Psychosis: A Delphi Study
Source: Front Psychiatry. 2021 Oct 22;12:760904. doi: 10.3389/fpsyt.2021.760904 (PMC8569300; doi:10.3389/fpsyt.2021.760904)
Supplement: Supplementary file 2 [file Table_1.docx]

**Supplementary Material A – Final group scores of all model of care attributes and levels**

Final groups scores of all model of care attributes and levels. Group mean scores were calculated to inform table listed order of attributes that received the same median score.

| **Theme** | **Question** | **Response options scored** | **Final Group score** (median or %) | **Final Group mean score** (where applicable) |
| --- | --- | --- | --- | --- |
| **PRE-RELEASE CARE PLANNING** | How important are these types of pre-release assessments in terms of leading to consumers getting appropriate care and support on release? | Clinical assessment | 5 | 4.81 |
|  |  | Social and Economic needs assessment | 4 | 4.05 |
|  |  | Reoffending/Community safety risk assessment | 4 | 3.86 |
|  |  | Demographic assessment (e.g., gender, age, cultural background) | 4 | 3.86 |
|  | You scored Clinical Assessment as an important pre-release assessment type. How important are the following elements to this assessment? | Active symptoms of psychosis or in remission | 5 | 5.00 |
|  |  | Comorbidity (e.g., substance use, PTSD) or intellectual/cognitive disability | 5 | 5.00 |
|  |  | Perceived risk to self or others | 5 | 4.95 |
|  |  | Functional Capacity | 4 | 4.15 |
|  | You scored Reoffending/Community Safety Risk Assessment as an important pre-release assessment type. How important are the following elements to this assessment? | Substance abuse | 5 | 5.00 |
|  |  | Emotional stability/Mental Health | 5 | 4.90 |
|  |  | Housing and financial stability | 5 | 4.65 |
|  |  | Peer relationships | 4 | 4.10 |
|  |  | Criminal orientation and thinking | 4 | 4.00 |
|  |  | Education/ Employment | 4 | 3.90 |
|  |  | Placed on Terrorism High Risk Offenders scheme | 4 | 3.90 |
|  |  | Parenting skills/Family relationships | 4 | 3.80 |
|  |  | Number of convicted offences | 3 | 3.15 |
|  |  | Leisure/Recreation involvement | 3 | 3.00 |
|  |  | Registered with the Serious Offenders Review Council (SORC) | 3 | 2.95 |
|  | You scored Demographic Assessment as an important pre-release assessment type. How important are the following elements to this assessment? | Aboriginal and/or Torres Strait Islander status | 4 | 4.05 |
|  |  | Other people with cultural and linguistic diverse backgrounds | 4 | 4.00 |
|  |  | Age (n.b. this excludes those aged under 18 years) | 4 | 3.95 |
|  |  | Gender | 4 | 3.90 |
|  |  | LGBTIQ+ status | 4 | 3.85 |
|  | You scored Social and Economic Assessment as an important pre-release assessment type. How important are the following elements to this assessment? | Housing | 5 | 4.95 |
|  |  | Financial/Income support | 4 | 4.10 |
|  |  | Family, Carer, Guardian support level | 4 | 4.10 |
|  |  | Employment, Vocational training, and Education | 4 | 4.00 |
|  |  | Peer relationships and Community involvement level | 4 | 4.00 |
|  | Exclusion Criteria: Should any of these specific groups be excluded from the Model of Care? | Those registered with the Serious Offenders Review Council (SORC), who typically include those: a) serving a sentence for life, or b) those serving a (or series of) sentence(s) with a non-parole period of 12 years | NO: 100.00% YES: 0.00% | |
|  |  | Those placed on the Terrorism High Risk Offenders (THRO) scheme | NO: 100.00% YES: 0.00% | |
|  |  | Those whose most recent sentence was for a violent offence (non-sexual) | NO: 100.00% YES: 0.00% | |
|  |  | Those whose most recent sentence was for a sexual or violent offence | NO: 100.00% YES: 0.00% | |
|  |  | Do you agree with the following statement: No one should be automatically excluded from the model of care – should consider all people on a case-by-case basis | AGREE: 95.24% DISAGREE: 4.76% | |
|  | Time of assessment: Ideally, when should assessments for post-prison care planning begin? | On entry to prison, then repeat periodically while in prison  Six to nine months prior to release  Three to six months prior to release  One to three months prior to release | On entry to prison, then repeat periodically while in prison: 95.24%  Six to nine months prior to release: 0.00%  Three to six months prior to release: 4.17%  One to three months prior to release: 0.00% | |
|  | Consumer motivation to engage in care plan development: How important are the following strategies to motivate consumers to engage in care planning? | Undertake motivational work in prison to ensure the consumer is engaged in care planning before release | 4 | 4.14 |
|  |  | Address engagement barriers that may occur in prison (e.g., mental health related stigma, consumer confidentiality issues, timely access to mental health services, fear of Risk Intervention Team (RIT) practices (e.g., safe cell/ isolation) | 4 | 4.05 |
|  |  | Mandate care plan if necessary (e.g., treatment order, parole condition) | 4 | 4.00 |
|  | Stakeholder involvement in care plan: How important is it to **INVOLVE**the following stakeholders in the development of a consumer's care plan? | NGO/Community organisation/Aboriginal-Community Controlled Health Organisation | 5 | 5.00 |
|  |  | Housing (Department of Communities & Justice) | 5 | 4.95 |
|  |  | The Mental Health Review Tribunal | 5 | 4.81 |
|  |  | Justice Health & Forensic Mental Health Network | 4 | 4.10 |
|  |  | Community Corrections | 4 | 4.05 |
|  |  | Consumer/carer/family member/guardian/peer | 4 | 4.00 |
|  |  | Correctional centre personnel | 4 | 3.86 |
|  |  | Police | 4 | 3.67 |
|  |  | Local Health District | 3 | 3.43 |
|  | How important is it for the following stakeholders to **LEAD** the development of a consumer's care plan? | Justice Health & Forensic Mental Health Network | 4 | 3.90 |
|  |  | Consumer/carer/family member/guardian/peer | 4 | 3.86 |
|  |  | Local Health District | 4 | 3.76 |
|  |  | Establish a new independent specialised service/ team for this model of care | 4 | 3.67 |
|  |  | NGO/Community organisation/Aboriginal-Community Controlled Health Organisation | 3 | 3.10 |
|  |  | Community Corrections | 3 | 2.90 |
|  |  | Housing (Department of Communities & Justice) | 3 | 2.86 |
|  |  | The Mental Health Review Tribunal | 3 | 2.71 |
|  |  | Correctional centre personnel | 3 | 2.57 |
|  |  | Police | 1 | 1.10 |
|  | Care plan coordination/ operationalising, including integration of services and treatments: Should a case manager be responsible for operationalising the care plan, including integration of services and treatments? | YES NO | YES: 100.00% NO: 0.00% | |
|  | Which agency/organisation should the case manager be affiliated to? | New independent specialised service/team created especially for this model of care  JH&FMHN  NGO/Community Organisation/Aboriginal Community-Controlled Health Organisation  Local Health District  The Mental Health Review Tribunal  Corrective services  Carer/Family member/Guardian/ Peer  Housing (Department of Communities & Justice)  Police | New independent specialised service/team created especially for this model of care: 71.43%  JH&FMHN: 14.29%  NGO/Community Organisation/Aboriginal Community-Controlled Health Organisation: 4.76%  Local Health District: 4.76%  The Mental Health Review Tribunal: 0.00%  Corrective services: 4.76%  Carer/Family member/Guardian/Peer: 0.00%  Housing (Department of Communities & Justice): 0.00%  Police: 0.00% | |
|  | Information sharing between services: Should there be information sharing between all agencies working with the consumer? | Yes. There should be in place a multi-agency working agreement outlining agreed information-sharing procedures including confidentiality provisions  No. There should not be any information sharing between agencies | Yes. There should be in place a multi-agency working agreement outlining agreed information-sharing procedures including confidentiality provisions: 95.24%  No. There should not be any information sharing between agencies: 4.76% | |

| **Theme** | **Question** | **Response options scored** | **Final Group score** (median or %) | **Final Group mean score** (where applicable) |
| --- | --- | --- | --- | --- |
| **TREATMENT** | Which of the following case management approaches would best suit consumers with **lower level/primary health care needs?** | 1. Case manager helps the consumer to identify their care needs and helps them access, navigate and interact with services in the community, including general practitioners (GPs), allied health professionals, pharmacists, Aboriginal health practitioners, and housing and income support providers (traditional brokerage/referral model of case management)  2. Case manager is part of a specialised team which identifies the consumer’s care needs and directly delivers all treatments and services, including social and economic supports to the consumer (assertive community treatment model)  3. Case manager is part of a specialised team which identifies the consumer’s care needs and directly delivers some treatments/services and helps the consumer access, navigate, and interact with other external services. (50/50 split of 1 & 2) | 1. Case manager helps the consumer to identify their care needs and helps them access, navigate and interact with services in the community, including general practitioners (GPs), allied health professionals, pharmacists, Aboriginal health practitioners, and housing and income support providers (traditional brokerage/referral model of case management): 14.29%  2. Case manager is part of a specialised team which identifies the consumer’s care needs and directly delivers all treatments and services, including social and economic supports to the consumer (assertive community treatment model): 0.00%  3. Case manager is part of a specialised team which identifies the consumer’s care needs and directly delivers some treatments/services and helps the consumer access, navigate, and interact with other external services. (50/50 split of 1 & 2): 85.71% | |
|  | Which of the following attributes are important to consider when planning a treatment model for consumers with **lower level/primary health care needs?** | Working with consumer’s informal support network (e.g., family, carers, landlords, teachers/employers, etc.) | 4 | 4.00 |
|  |  | Supervision arrangements for those assessed as a medium to high reoffending risk (CCO vs other case manager) | 4 | 3.86 |
|  |  | Forensic mental health training for treatment/service providers | 3 | 3.14 |
|  | You stated that '**supervision arrangements for those assessed as medium to high risk (reoffending)'** is an important treatment model attribute for consumers with lower level/ primary care needs. Who should ideally supervise these groups? | Supervision occurs by a Community Corrections Officer  Case manager who implements the consumer’s care plan (if case manager is not Community Corrections Officer) | Supervision occurs by a Community Corrections Officer: 70.00%   Case manager who implements the consumer’s care plan (if case manager is not Community Corrections Officer): 30.00% | |
|  | Which of the following case management approaches would best suit consumers with **medium to high level health care needs?** | 1. Case manager helps the consumer to identify their care needs and helps them access, navigate and interact with services in the community, including general practitioners (GPs), allied health professionals, pharmacists, Aboriginal health practitioners, and housing and income support providers (traditional brokerage/referral model of case management)  2. Case manager is part of a specialised team which identifies the consumer’s care needs and directly delivers all treatments and services, including social and economic supports to the consumer (assertive community treatment model)  3. Case manager is part of a specialised team which identifies the consumer’s care needs and directly delivers some treatments/services and helps the consumer access, navigate, and interact with other external services. (**50/50 split of 1 & 2**) | 1. Case manager helps the consumer to identify their care needs and helps them access, navigate and interact with services in the community, including general practitioners (GPs), allied health professionals, pharmacists, Aboriginal health practitioners, and housing and income support providers (traditional brokerage/referral model of case management): 4.76%  2. Case manager is part of a specialised team which identifies the consumer’s care needs and directly delivers all treatments and services, including social and economic supports to the consumer (assertive community treatment model): 4.76%  3. Case manager is part of a specialised team which identifies the consumer’s care needs and directly delivers some treatments/services and helps the consumer access, navigate, and interact with other external services. (**50/50 split of 1 & 2**): 90.45% | |
|  | **You selected option 2 or 3.** For this option, **which of the following attributes are important** to consider when planning a treatment model for consumers with **medium to high level health care needs?** | Specialised team composition | 5 | 5.00 |
|  |  | Treatment accessibility | 5 | 5.00 |
|  |  | Specialised team leader | 4 | 4.10 |
|  |  | Contact frequency with consumer | 4 | 4.10 |
|  |  | 24-hour psychiatric crisis service | 4 | 4.10 |
|  |  | Case load of case manager | 4 | 4.05 |
|  |  | Treatment type | 4 | 4.05 |
|  |  | Agency delivering each treatment type | 4 | 4.05 |
|  |  | Location of treatments (Community outreach vs treatment provider clinic/office) | 4 | 4.05 |
|  |  | Specialised team are closely involved in decisions to admit to hospital care and hospital discharge planning | 4 | 4.05 |
|  |  | Specialised team works with consumers' informal support network (e.g., family, carers, landlords, teachers/employers, etc) | 4 | 4.00 |
|  |  | Forensic mental health expertise/training for treatment/service providers | 4 | 4.00 |
|  |  | Supervision arrangements for those assessed as medium to high reoffending risk (CCO vs other case manager) | 4 | 4.00 |
|  | You stated that '**specialised team composition**' is an important treatment model attribute for consumers **with medium to high level care needs**.  How important is it to have the following **staff as part of the treatments team**? | Psychiatrist | 5 | 4.76 |
|  |  | Nurse | 4 | 4.14 |
|  |  | Substance abuse specialist | 4 | 4.10 |
|  |  | Social worker | 4 | 4.05 |
|  |  | Psychologist | 4 | 4.00 |
|  |  | Aboriginal health practitioner | 4 | 3.95 |
|  |  | Social support service provider (NGO) | 4 | 3.90 |
|  |  | Housing provider | 4 | 3.90 |
|  |  | Peer support worker | 4 | 3.86 |
|  |  | Vocational/training specialist | 4 | 3.76 |
|  |  | Diet/nutrition specialist | 3 | 2.90 |
|  |  | Probation and parole/CCO | 3 | 2.90 |
|  |  | Exercise specialist | 3 | 2.90 |
|  |  | Police | 2 | 1.86 |
|  | You stated that **'treatment accessibility**' is an important treatment model attribute for consumers **with medium to high level care needs**. For treatments that are not delivered at the consumer's home through outreach, how important are the **following strategies to improve access to treatments**? | Tele-health service using audio-visual link on computer | 4 | 4.05 |
|  |  | Transport support to attend treatment/service | 4 | 3.90 |
|  |  | Tele-health service using audio-visual link on phone | 3 | 3.43 |
|  | You stated that '**specialised team composition**' is an important treatment model attribute for consumers **with medium to high level care needs.** How important is it to have the following staff **as the leader of the treatments team**? | Psychiatrist | 5 | 4.65 |
|  |  | Nurse | 4 | 3.75 |
|  |  | Psychologist | 4 | 3.65 |
|  |  | Social worker | 4 | 3.60 |
|  |  | Social support service provider (NGO) | 3 | 3.00 |
|  |  | Aboriginal health practitioner | 3 | 2.90 |
|  |  | Peer support worker | 3 | 2.85 |
|  |  | Substance abuse specialist | 3 | 2.80 |
|  |  | Housing provider | 2 | 2.10 |
|  |  | Vocational/training specialist | 2 | 1.90 |
|  |  | Exercise specialist | 2 | 1.90 |
|  |  | Diet/nutrition specialist | 2 | 1.90 |
|  |  | Probation and parole/CCO | 2 | 1.85 |
|  |  | Police | 1 | 1.00 |
|  | You stated that **'contact frequency with the consume**r' is an important treatment model attribute for consumers with **medium to high level care needs.** How many times per week should team members be **in face-to-face contact with the consumer**? | Once or twice a week  Average of less than 1 face to face contact per week  Three to four times per week | Once or twice a week: 90.48%  Average of less than 1 face to face contact per week: 9.52%  Three to four times per week: 0.00% | |
|  | You stated that **'24-hour psychiatric crisis service**' is an important treatment model attribute for consumers with **medium to high level care needs**. How important are the following **arrangements for 24-hour psychiatric crisis service**? | The specialised team is notified of crisis through the general crisis line of community mental health service | 4 | 3.86 |
|  |  | The specialised team are the first line of crisis intervention (i.e., a team member is always on-call) | 3 | 3.19 |
|  | You stated that **'case load of the case manager**' is an important treatment model attribute for consumers with **medium to high care needs.** What is an **ideal maximum case load?** | 10 or fewer consumers per case manager  11-20 consumers per case manager  21-34 consumers per case manager  35-49 consumers per case manager  50 or more consumers per case manager | 10 or fewer consumers per case manager: 71.43%  11-20 consumers per case manager: 19.05%  21-34 consumers per case manager: 4.76%  35-49 consumers per case manager: 4.76%  50 or more consumers per case manager: 0.00% | |
|  | You stated that '**'treatment type'** is an important treatment model attribute for consumers with **medium to high level care needs**. While every consumer will have different treatment type needs, how important is it to consider each of the following treatments? | Psychological support/counselling | 5 | 4.95 |
|  |  | Drug and alcohol support | 5 | 4.95 |
|  |  | Independent living skills support | 5 | 4.95 |
|  |  | Pharmacotherapy | 5 | 4.86 |
|  |  | Social and economic supports | 5 | 4.81 |
|  |  | Residential rehabilitation | 4 | 4.05 |
|  |  | Diet/nutrition support | 3 | 3.00 |
|  |  | Exercise support | 3 | 3.00 |
|  | You stated that '**agency delivering treatment type**' is an important treatment model attribute for consumers with **medium to high level care needs.** Which of the following agency/organisation/position is best suited to deliver each treatment type? | Psychological support/counselling | New independent specialised service/team: 76.19%  Local Health District: 9.52%  NGO/Community Organisation/Aboriginal community-controlled health organisation: 9.52%  JH&FMHN: 4.76%  Community Corrections: 0.00% | |
|  |  | Drug and alcohol support | New independent specialised service/team: 71.43%  NGO/Community Organisation/Aboriginal community-controlled health organisation: 19.05%  Local Health District: 9.52%  Community Corrections: 0.00%  JH&FMHN: 0.00% | |
|  |  | Independent living skills support | NGO/Community Organisation/Aboriginal community-controlled health organisation: 100.00%  New independent specialised service/team: 0.00%  Local Health District: 0.00%  JH&FMHN: 0.00%  Community Corrections: 0.00% | |
|  |  | Pharmacotherapy | Local Health District: 80.95%  New independent specialised service/team: 14.20%  NGO/Community Organisation/Aboriginal community-controlled health organisation: 4.76%  Community Corrections: 0.00%  JH&FMHN: 0.00% | |
|  |  | Social and economic supports | NGO/Community Organisation/ Aboriginal community-controlled health organisation: 95.24%  New independent specialised service/team: 0.00%  Local Health District: 4.76%  Community Corrections: 0.00%  JH&FMHN: 0.00% | |
|  |  | Residential rehabilitation | NGO/Community Organisation/Aboriginal community-controlled health organisation: 100.00%  Local Health District: 0.00%  New independent specialised service/team: 0.00%  JH&FMHN: 0.00%  Community Corrections: 0.00% | |
|  |  | Diet/nutrition support | Local Health District: 95.24%  NGO/Community Organisation/Aboriginal community-controlled health organisation: 4.76%  New independent specialised service/team: 0.00%  Community Corrections: 0.00%  JH&FMHN: 0.00% | |
|  |  | Exercise support | NGO/Community Organisation/Aboriginal community-controlled health organisation: 90.48%  Local Health District: 9.52%  New independent specialised service/team: 0.00%  Community Corrections: 0.00%  JH&FMHN: 0.00% | |
|  | You stated that **'location of treatments**' is an important treatment model attribute for consumers with **medium to high level care needs.** For people not requiring hospital care, how important is it to have treatments/services delivered at the following locations? | Treatments/services should be mostly outreach-based: delivered in the community where a consumer lives or interacts with other people rather than at an office  Treatments/services should be mostly based at the office location of the treatments team  Treatments/services should be split 50/50 between outreach in community and based at the office location of the treatments team | Treatments/services should be mostly outreach-based: delivered in the community where a consumer lives or interacts with other people rather than at an office: 28.57%  Treatments/services should be mostly based at the office location of the treatments team: 0.00%  Treatments/services should be split 50/50 between outreach in community and based at the office location of the treatments team: 71.43% | |
|  | You stated that **'supervision arrangements for those assessed as medium to high reoffending risk'** is an important treatment model attribute for consumers with **medium to high level care needs**. **Who should ideally supervise** this group? | Case manager who implements the consumer’s care plan (if case manager is not Community Corrections Officer)  Supervision occurs by a Community Corrections Officer | Case manager who implements the consumer’s care plan (if case manager is not Community Corrections Officer): 80.95%  Supervision occurs by a Community Corrections Officer: 19.05% | |
|  | You stated that **'forensic mental health experience/training for specialised team**' was an important treatment model attribute for consumers with **medium to high level care needs.** How important is it for the following staff to have forensic mental health training or expertise? | Psychiatrist | 5 | 4.95 |
|  |  | Nurse | 5 | 4.95 |
|  |  | Psychologist | 5 | 4.75 |
|  |  | Substance abuse specialist | 4 | 4.05 |
|  |  | Probation and parole/CCO | 4 | 4.00 |
|  |  | Aboriginal health practitioner | 4 | 3.95 |
|  |  | Social worker | 4 | 3.90 |
|  |  | Social support service provider (NGO) | 4 | 3.90 |
|  |  | Peer support worker | 4 | 3.85 |
|  |  | Vocational/training specialist | 4 | 3.80 |
|  |  | Housing provider | 4 | 3.80 |
|  |  | Police | 3 | 3.00 |
|  |  | Exercise specialist | 3 | 2.95 |
|  |  | Diet/nutrition specialist | 2 | 2.00 |

| **Theme** | **Question** | **Response options scored** | **Final Group score** (%) |
| --- | --- | --- | --- |
| **DIVERSION FROM PRISON** | Do you agree/disagree with the following statements?? | Diversion from prison is the default option for all consumers until the prosecution convinces the court otherwise | AGREE: 85.71% DISAGREE: 14.29% |
|  |  | Diversion from prison is conditional on the crime committed and/or type of parole breach | AGREE: 90.48% DISAGREE: 9.52% |
|  |  | A treatment and care plan should be made available for those not diverted and return to prison | AGREE: 100.00% DISAGREE: 0.00% |
|  |  | Consumers diverted from prison should have their care plan reviewed | AGREE: 100.00% DISAGREE: 0.00% |
|  |  | Court diversion should be available to all consumers (regardless of residential address) | AGREE: 100.00% DISAGREE: 0.00% |

| **Theme** | **Question** | **Response options scored** | **Final Group**  **score** (median) | **Final Group mean score** |
| --- | --- | --- | --- | --- |
| **EVALUATION** | How important are the following attributes of Evaluation? | Effectiveness in managing psychosis and comorbidity | 5 | 4.76 |
|  |  | Effectiveness in reducing reoffending | 4 | 4.29 |
|  |  | Consumer Satisfaction | 4 | 4.19 |
|  |  | Social and Economic outcomes | 4 | 4.10 |
|  |  | Health Economics Evaluation | 4 | 4.10 |
|  |  | Successful Integration of Services | 4 | 3.95 |
